# Effects of Abnormal Oral Reflexes on Speech Articulation in Persian Speaking Children with Spastic Cerebral Palsy

**Published:** 2016

**Authors:** Hooshang DADGAR, Mohammad Reza HADIAN, Ortega Adriana LIRA

**Affiliations:** 1 Department of Speech Therapy, School of Rehabilitation, Tehran University of Medical Sciences, Tehran, Iran.; 2 Department of Post Graduate Studies, Brain and Spinal injury Research Center (BASIR), School of Rehabilitation, Tehran University of Medical Sciences, International Campus (TUMS,TUMS-I), Tehran, Iran; 3 Specialist in Pediatric Dentistry and in Dentistry for Special Needs Pacients, Coordinator Professor of the Specialization Course in Pediatric Dentistry at Dentistry School Foundation (FFO) of the University of São Paulo, Brazil.

**Keywords:** Cerebral palsy, Abnormal oral reflexes, Sound articulation, Persian

## Abstract

**Objective:**

The purpose of this study was to investigate the relationship between the presence of abnormal oral reflexes and speech sound production in children with severe cerebral palsy.

**Materials & Methods:**

Seven oral reflexes such as, rooting, mouth-opening, biting, chewing, lip, tongue, and suckling were examined in 52Persian-speaking monolingual children with spastic cerebral palsy (ages 5-10 yr).Phonetic information tests were administered to investigate their ability for articulation of the speech sounds.

**Results:**

A significant relationship between three (i.e. the chewing, lip, and biting reflexes) out of the seven abnormal oral reflexes and the speech articulation was noticed. The presence of the chewing reflex was associated with deficits in production of /s, z, š,č/ sounds. The lip reflex was associated with deficits in the production of /p, m, r, j, f, č/ sounds. The biting reflex was associated with deficits in the production of /z, l, y and š/ sounds. No significant relationship was found between the rooting, mouth-opening, tongue, and suckling reflexes and sound articulation.

**Conclusion:**

The presence of abnormal reflexes in the children with spastic cerebral palsy would suggest a correlation between these reflexes and sound articulation in Iranian children with spastic cerebral palsy. Hence, these observations might suggest some disturbances in normal speech development.

## Introduction

There are three different types of Cerebral palsy (CP) as spastic, ataxic, and athetosis (1-3). It is mainly a movement disorder caused by non-progressive brain damage which occurs during the fetal development and birth. The prevalence of CP in children between 3-10 yr is 2.4/1000 live-born children (4-6). Classification of CP has also been made by the numbers of limbs that are affected (5, 7, 8) and the most common type is spastic CP (3, 4,7). CP is commonly associated with the disorders of vision, hearing, speech, receptive and expressive language function, swallowing/feeding, and oral motor function (9, 10). Oral motor disorders in CP include jaw instability, tongue thrusting, poor tongue movement, poor lip closure,and excessive drooling. Abnormal oral reflexes have also been observed in children with CP (11). Clinically, the oral reflexes are not usually observed in children after 18 months (12); therefore, their developments are quite critical for normal feeding, nutrition, and speech. The persistence of these reflexes after 18 months in children with CP is considered abnormal and may have negative effects on the oral motor functions. There are few studies regarding the abnormal oral reflexes in children with CP and their association with speech problems (12, 13). Accordingly, Sheppard (1964) reported a relation between the primitive craniooropharyngeal motor patterns and speech performance in athetosis and spastic types of CP (14). Seven abnormal oral reflexes or deviant oral behavior characteristics have been reported in children with CP as follows: rooting, mouth-opening, biting, chewing, lip, tongue, and suckling (12-14). Some speech and language pathologists argue that the presence of abnormaloral reflexes in children older than 2 yr with CP may indicate the immature or pharyngeal functions. Poor oral function can contribute to some problems in speech articulation. The main object of this study was to investigate the relationship between the presence of abnormal oral reflexes and articulation in Iranian children with spastic CP.

## Materials & Methods

**Participants**

In this non-interventional study, 52 children with spastic CP (5 to 10 yr, ±8.5 - SD 0.0565) referred for rehabilitation services to the clinic of the Faculty of Rehabilitation (Tehran University of Medical Sciences, Tehran, Iran) enrolled in the study. Inclusion criteria were based on medical and rehabilitation records and also by the assessments of speech and language pathologist. The participant had to have no prior records for language deficit, intellectual deficit, auditory or visual problems, or craniofacial deformities such as a cleft palate. All participants were monolingual native speakers of Persian. The ethical committee of Tehran University of Medical Sciences approved the study and a written informed consent formed was obtained from all parents.

**Procedures**

Oral reflexes such as rooting, opening of the mouth, lip, tongue, and biting, chewing and suckling reflexes were evaluated through direct observations and assessments of the children by a speech and language pathologist. Assessments were based on the presence or absence of reflexes through the reactions of children to stimuli as described by Mysak (12, 13; Table 1).

**Table 1:** Reflexes Assessment According Mysak and Sheppard.

**Type of reflex**	**Stimulus**	**Response**
Rooting reflex	Touching the corner of lip, philtrum in the superior lip, and the middle of lower lip with the forefinger	Rotation of head, lip, tongue or mandible toward the direction of stimulation.
Suckling reflex	Slight pressure with a rotary movement to the corner of the mouth	Backward– forward lingual movements, such as suction movements
Biting reflex	Placing tongue depressor between the upper and lower teeth.	Upward jaw movement into a strongly clenched posture
Lip reflex	Tapping proximally from mid-cheek to the corner of the mouth	Alternate, rapid, puckering and extension of the lips.
Mouth-opening reflex	Moving two fingers in front of the mouth within the visual range of the subject.	Hyper-extension of the mandible or a lesser degree of mouth opening.
Chewing reflex	Rubbing the anterior and lateral surfaces of the teeth with a tongue depressor.	Flexion and extension of the jaw similar to chewing motion.
Tongue reflex	Touching of lateral margin of the tongue by finger	Tongue protrudes or Lateral tongue motion

**Table 2:** The Relationship between the Lip Reflex with Articulations of Bilabial and Labio-Dental Sounds in Iranian Children with Spastic Cerebral Palsy (N=52).

** Reflex**		**Lip reflex**		
**Constant**		**Normal**	**Abnormal**	***P*** **-value**
b	Accurate articulation	29	9	0.386
	Inaccurate articulation	9	5	
p	Accurate articulation	32	6	0.003
	Inaccurate articulation	6	8	
m	Accurate articulation	36	10	0.038 *
	Inaccurate articulation	2	4	
v	Accurate articulation	20	4	0.123 *
	Inaccurate articulation	18	10	
f	Accurate articulation	30	4	0.001 *
	Inaccurate articulation	8	10	

* Fisher's Exact was employed because small expected values encountered.

**Table 3:** The Relationship between Biting and Chewing Reflexes with Articulation in Iranian Children with Spastic Cerebral Palsy (N=52).

** Reflex**		**Biting reflex**			**Chewing reflex**		
**Constant**		**Normal**	**Abnormal**	***P*** **-value**	**Normal**	**Abnormal**	***P*** **-value**
t	Normal articulation	22	13	0.373	9	25	1.000 *
	Abnormal articulation	8	9		4	4	
d	Normal articulation	25	12	0.065	11	27	0.472 *
	Abnormal articulation	5	9		2	12	
n	Normal articulation	25	19	1.000 *	9	25	0.096 *
	Abnormal articulation	5	2		4	4	
l	Normal articulation	21	9	0.049	7	22	0.757
	Abnormal articulation	9	13		6	16	
s	Normal articulation	16	7	0.162	10	13	0.009 *
	Abnormal articulation	14	15		3	26	
z	Normal articulation	16	4	0.020 *	9	11	0.019 *
	Abnormal articulation	14	18		4	28	
j	Normal articulation	20	6	0.011	9	17	0.199 *
	Abnormal articulation	10	16		4	22	
r	Normal articulation	9	5	0.753	9	5	0.752
	Abnormal articulation	21	17		21	17	
ʃ	Normal articulation	17	2	0.002 *	10	10	0.002 *
	Abnormal articulation	12	19		3	29	
ž	Normal articulation	8	4	0.526 *	3	9	1.000 *
	Abnormal articulation	22	18		10	30	
č	Normal articulation	20	10	0.161	0	30	0.004 *
	Abnormal articulation	10	12		6	16	

* Fisher's Exact was employed because small expected values encountered.

**Fig 1:**
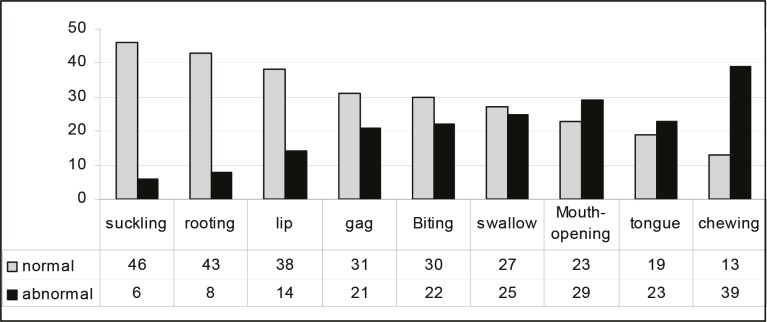
Position of oral reflexes in Iranian children with spastic cerebral palsy (N=52)

**Fig 2:**
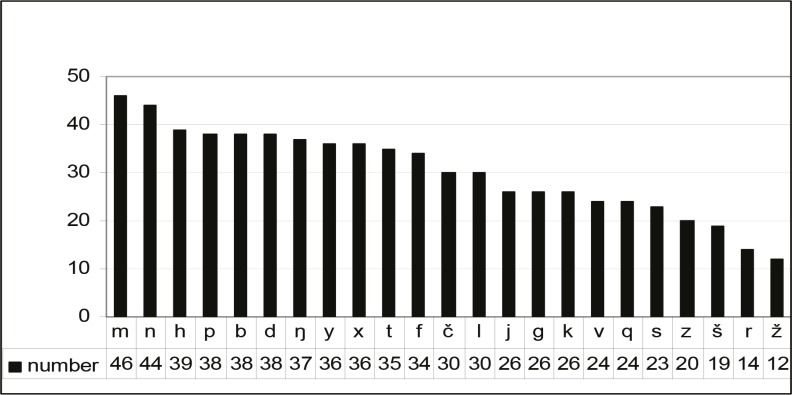
State of normal articulation at all positions in Iranian children with spastic cerebral palsy (N=52).

The Persian language comprise of twenty-three consonants and six vowels. According to the manner of articulation, Persian consonants contain the seven plosives/ p, b, g, k, t, d,q/,eight fricatives/f, v, s, z, h, x, š, ž /, three nasals/m,ŋ, n/, two affricates/ č, j /one trill /r/, one sound that is an approximant/y/, and one lateral approximant/l/(15). Most Persian children can correctly produce all consonants by the age of 5 yr (16). Sixty-eight pictures were presented as a phonetic test to make assessments for articulation. The test consisted of Persian consonants being placed at initial, middle and final positions in words. Children were asked to name these words and then, any error in articulation was recorded on a tape by a speech and language pathologist. A speech sound was considered as an error if it was misarticulated in at least one word position and in at least one word. Substitutions, omissions and distortions were considered as errors. A speech-recorded sample from each child was transcribed using the International Phonetic Alphabet (IPA). The numbers of incorrect phonemes (extracted from the transcriptions) were determined for each child. All of the oral reflexes were assessed and those reflexes contributed to the articulation of the specific sounds such as the lip reflex for labial and labiodental sounds such as p and fare characterized in Table 2.

**Statistical analysis**

The Chi-squared test was used for statistical analysis in order to compare the relationship between the abnormal oral reflexes and articulations for speech sounds. Fisher’s Exact was employed for small expected values (less than 5) and the significance value was set at P<0.05.

## Results

Fifty-two children aged 5-10 yr with spastic CP participated in this study; comprising of 24 quadriplegia, 19 diplopia, 5 hemiplegia and 4 paraplegia. Evaluation for the presence of abnormal oral reflexes in children is shown in Figure1. The status of articulation according to the place and manner of articulations is shown in Figure2. The relationship between the presence of lip reflex and the articulation of sounds /p, m, r, y, f, č/is shown in Table 2. The presence of a chewing reflexing relationto the articulations of/z, š/ was significant (P<0.05; Table 3). There was no significant relationship between the presence of tongue and rooting reflexes with the articulation of any sound.

## Discussion

The purpose of this study was to evaluate the presence of abnormal oral reflexes and their possible effects on the ability of producing speech sounds in children with spastic CP. The results of this study showed that abnormal oral reflexes were common in older children with spastic CP in agreement with previous studies (12-14, 17).Our study also showed that the chewing reflex was the most common reflex observed in these children. However, the biting reflex was the most frequent abnormal reflex (18). Reflexes of suckling and rooting were the least frequent reflexes; in agreement with Love et al. study (18). In terms of the manner of articulation, the articulation of fricatives was relatively more difficult in children participated in this study; similar to Platt et al. results (19). Similarly, linguo-alveolar fricatives were closely linked with oral reflexes (20). However, dental and glottal sounds were the most problematic for these children during articulation (21). Our results revealed that the accuracy of articulation was the greatest for bilabial and nasal sounds; which were similar to the findings in normal children (22). There was a significant relationship between the presence of lip reflex and the articulation of sounds /p, m/;this could be explained by the variety of involuntary lip movements displayed by children with spastic CP. As the lip reflex might also trigger other oral movements such as tongue movement, misarticulation of the sounds i /r, y, f, č/ might also be observed in these children. Similar results were also reported by Mysak regarding the abnormal lip reflex and the possible interference with articulation of /p, m, f/ sounds (12). However, no relationship was reported between the oral reflexes and articulation proficiency in older CP children in another study (18). We saw a relationship between the biting and chewingreflexes and misarticulation of the sounds /z, š/. These observations might be due to the unregulated closing or opening of the jaw following the biting and chewing reflexes that could effect on tongue movements and this in turn could lead to the misarticulation of the sounds /z, š/. Some researchers have acclaimed this suggestion (12-14, 23); whereas others believed that the levels of oral reflexes and motor control in speech in the nervous system were separate mechanisms (24, 25). Therefore, suggested that the abnormal (primitive) oral reflexes had no effects on speech development. Our data showed an interrelation between the oral reflexes and sound articulation in older CP children. In addition, normal oral reflexes in CP play a primary role in feeding skills (12-14). Therefore, a possible relationship could be presumed between the development of oral reflexes and misarticulation of sounds in CP children. However, considering some controversies in studies, further studies are needed to elucidate these points. In addition, future studies could be carried out in order to investigate the presence of oral reflexes in different types of CP and also to determine the results of treatment of abnormal oral reflexes and their effects on sound articulation.

**In conclusion,** our results showed the presence of abnormal oral reflexes in children with CP and the possible effects of these reflexes on sound articulation.
